# Phase Nanoscopy with Correlated Frequency Combs

**DOI:** 10.3390/s23010301

**Published:** 2022-12-28

**Authors:** Xiaobing Zhu, Matthias Lenzner, Jean-Claude Diels

**Affiliations:** 1 School of Optical Science and Engineering, University of New Mexico, Albuquerque, NM 87106, USA; 2 Center for High Technology Materials, University of New Mexico, Albuquerque, NM 87106, USA; 3 Lenzner Research, 125 E Canyon View Dr, Tucson, AZ 85704, USA

**Keywords:** intracavity phase interferometry, laser sensors, precision sensing, inertial sensors, gyroscopes, ultrafast

## Abstract

This study addresses any sensor based on measuring a physical quantity through the phase of a probing beam. This includes sensing of rotation, acceleration, index change, displacement, fields… While most phase measurements are made by detecting an amplitude change in interfering beams, we detect instead a phase change through a relative frequency shift of two correlated frequency combs. This paper explores the limit sensitivity that this method can achieve, when the combs are generated in an Optical Parametric Oscillator (OPO), pumped synchronously by a train of femtosecond pulses separated by half the OPO cavity round-trip time. It is shown that a phase difference as small as 0.4 nanoradians can be resolved between the two pulses circulating in the cavity. This phase difference is one order of magnitude better than the previous record. The root-mean-square deviation of the measured phase over measuring time is close to the standard quantum limit (phase-photon number uncertainty product of 0.66). Innovations that made such improved performances possible include a more stable OPO cavity design; a stabilization system with a novel purely electronic locking of the OPO cavity length relative to that of the pump laser; a shorter pump laser cavity; and a square pulse generator for driving a 0.5 mm pathlength lithium niobate phase modulator. Future data acquisition improvements are suggested that will bring the phase sensitivity exactly to the standard quantum limit, and beyond the quantum limit by squeezing.

## 1. Introduction

**Figure 1:**
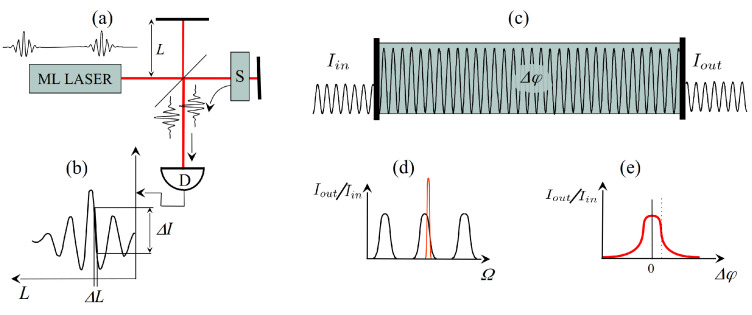
Passive interferometry. (**a**) the Michelson; (**b**) the phase detection proceeds via an amplitude measurement of interfering beams; (**c**) sensitivity enhancement using a high-Q resonator; (**d**) Fabry–Perot resonances vs. optical frequency. The higher the finesse, the narrower the transmission peaks. For optimum sensitivity, the laser linewidth should be narrower than the transmission peaks. (**e**) A phase shift Δφ within the interferometer (initially at resonance) results in a drop in transmission.

Sensors can provide information on a physical quantity through either amplitude or phase measurement. In general, even when phase information is to be extracted, the measurement proceeds via an intensity measurement. Let us consider the simple example of the Michelson interferometer as sketched in Figure 1a. The beam from a laser source (in the case of the figure, a mode-locked frequency comb) is split between a reference and sample arm. The purpose is to “sense” a change in optical path ΔL caused by the sample *S*. This change is measured as an intensity change in the interference pattern detected at the output of the Michelson (Figure 1b). The smallest detectable change in optical path ΔL is limited by amplitude noise of the source (and ultimately by photon noise). Even though the final measurement is that of a phase Δφ=kΔL, it proceeds via an amplitude measurement.

The phase resolution can be considerably improved by inserting the sample in a high-Q resonator. This technique has been exploited to the extreme by Ma et al. [1]. In that experiment, a high finesse ($10^{5}$) Fabry–Perot containing the sample to be analyzed is irradiated in transmission (Figure 1c). The Fabry–Perot transmission is sketched as a function of optical angular frequency Ω in Figure 1d. A change in phase (or amplitude) of the intracavity sample results in a drop in transmission. Optimum sensitivity calls for a laser linewidth narrower than the linewidth of the Fabry–Perot (red line in Figure 1d), in order to have the steepest drop in transmission with phase change Δφ (Figure 1e). Implementing the high cavity finesse combined with a laser linewidth of the order of 1 Hz is a challenge requiring sophisticated electronic stabilization [1]. Here, again, the phase determination Δφ proceeds via an amplitude measurement, limited by shot noise.

**Figure 2:**
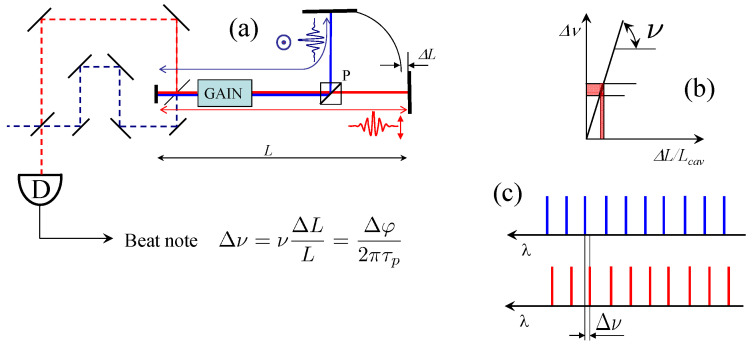
Active interferometry. (**a**) the 2 pulse/cavity mode-locked laser. The two pulses are orthogonally polarized, and split into two branches in a Michelson-like end-cavity interferometer. The two pulses extracted from the cavity are made to interfere via a beating interferometer. The *frequency* of the beat signal is a measure of the elongation ΔL. (**b**) The slope of the response Δν(ν) is the optical frequency ν. (**c**) The detector *D* records the interference of two frequency combs, with a 100% visibility.

The Michelson interferometer becomes an *active* sensor if inserted in a laser cavity as sketched in Figure 2. Generally speaking, we define as active a sensor which is inserted in an optical oscillator, such as a laser or an optical parametric oscillator. It has long been recognized that the output spectrum of a laser is extremely sensitive to the presence of an intracavity absorbing element [2]. It is the sensitivity of the laser mode frequency to an intracavity phase perturbation that is exploited here. In the example chosen in Figure 2, the laser is mode-locked. At one end of the cavity, a polarizing beam splitter provides two paths for two orthogonal polarization orientations. In one of the two arms, the pulse will be subject to a phase shift caused by the physical quantity to be measured. Except for this small section, the two pulses share the same gain medium and cavity (Figure 2a). If the gain is provided by synchronously pumped parametric oscillation, the two circulating pulses will have the same group round-trip time $\tau_{rt}$. The optical length *L*—hence the mode spacing—of the two cavities is slightly different, resulting in a different *phase* round-trip time $\tau_{p}=2Ln_{cav}/c$, where $n_{cav}$ is the index of refraction averaged over the cavity. Therefore, the two pulses circulating in the cavity—in the absence of coupling—will have slightly different frequencies. The difference can be measured as a beat note of frequency given by:

(1) $$\Delta \nu \;=\;\nu \frac{\Delta L}{L}\;=\;\frac{\Delta \varphi }{2\pi \tau_{p}}$$

where ΔL is the difference in optical path, and Δφ=kΔL the corresponding phase difference seen by the two pulses at each cavity round-trip. Note that the beat frequency Δν is equal to the relative elongation scaled by the optical frequency ν (Figure 2b). This poses stringent requirements on the stability of the end-cavity interferometer, since, for a typical 1-m cavity, a pm displacement will already produce a beat note in the kHz range. The two outputs of the laser are two frequency combs of the same tooth spacing. Since the tooth spacing of frequency combs is constant over the spectrum [3,4,5], the interference signal has near 100% visibility (Figure 2c).

## 2. Plurality of Sensors

**Figure 3:**
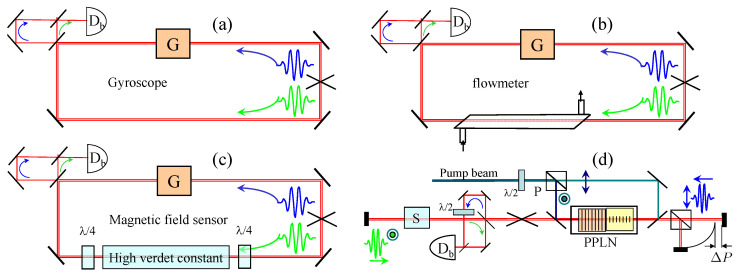
Examples of sensors based on active interferometry. (**a**) The laser gyroscope; (**b**) flowmeter application [8]; (**c**) magnetometer [9]; (**d**) linear configuration: the gain is provided by two orthogonal PPLN. P = polarizing beam splitter. The signal to be detected can be from a sample S of unknown dichroism or a length imbalance ΔP of an end-cavity interferometer.

Active interferometry can be applied to any sensor where the physical quantity to be measured can result in a differential phase between the two intracavity pulses. Some examples are illustrated in Figure 3. The first three figures show examples of ring laser sensors, while the bottom right figure shows an example of a sensor using a linear cavity. The most common form of intracavity interferometry is the laser gyroscope, where the phase difference per round-trip is the Sagnac phase shift [6,7]:

(2) $$\Delta \varphi_{s}\;=\;\frac{8\pi A}{c\lambda }\;\Omega_{R}.$$

where *A* is the area of the loop, and $\Omega_{R}$ is the angular rotation velocity around an axis orthogonal to the loop area. The phase shift leads to a beat note of:

(3) $$\Delta \nu \;=\;\frac{\Delta \varphi_{s}}{2\pi \tau_{p}}\;=\;\frac{4A}{\lambda P}\Omega_{R},$$

where *P* is the perimeter of the cavity. The rotation is the only response of which the sensitivity is proportional to the linear dimensions. All other responses are *inversely* proportional to the linear dimensions, favoring miniaturization. The first demonstration of flow measurement by intracavity phase interferometry [8] involved the arrangement of Figure 3b. Magnetic field measurements are often based on a Faraday rotation measurement, using a high Verdet constant material (for instance, a resonant atomic vapor in the case of the atomic magnetometer). Faraday rotation is nothing else than a difference in phase shift between right and left circular polarization in a magnetic field. Direct measurement of the phase shift by active interferometry (as in the setup of Figure 3c) is considerably more sensitive than the traditional rotation of linearly polarized light [9]. As will be shown in Section 3.4, a phase resolution better than 1 nanoradian is possible. To our knowledge, a nanoradian resolution in Faraday rotation has never been achieved.

Linear configurations are typically easier to implement. Figure 3d is an example of a Parametric Optical Oscillator (OPO) containing two orthogonally polarized pulses in the cavity. An element marked “sample” with minuscule dichroism will produce a measurable beat note. Having the two orthogonally polarized pulses separated at the cavity end as in Figure 3d offers as varied applications as the Michelson interferometer, with enhanced signal to noise.

The various applications have been discussed in review articles [10,11]. In most cases, the resolution is limited by noise in the sensor element (for instance, mechanical vibrations in the arms of a Michelson interferometer). This work aims at finding the intrinsic resolution of Intracavity Phase Interferometry, independently of the application. It will be shown in Section 3.3 that, with an end cavity interferometer of ultimate rigidity, a sub-femtometer resolution is possible, making it possible to monitor indices of refraction, layer thicknesses, stresses, etc… A stiff inertial mass in one branch makes this device an accelerometer. The possibility of having a dead band created by backscattering at the pulse crossing point is even reduced by having the two pulses orthogonally polarized.

## 3. Limits of Noise

### 3.1. Noise in Elongation Measurement

The OPO is often the preferred configuration for Intracavity Phase Interferometry [12,13]. There is a lower and upper limit for the range in optical path ΔP that can be measured in the arrangement of Figure 3d. The maximum displacement is such that the displacement is of the order of the mode spacing, or ΔP≈λ/2. The smallest measurable displacement depends on the mechanical noise in the interferometer. This noise can be reduced by using bigger, stable mirror masses, shorter optical paths (eventually in vacuum), more compact and stiffer structures. There is, however, a quantum limit to the interferometer’s stability, as demonstrated by Caves [14]. This “dog-that-bites-its-tail” demonstration is briefly summarized below. From the position–momentum uncertainty relation ΔxΔp≥ℏ/2, one extracts the minimum momentum $\Delta p_{min}=\hbar /\left(2\Delta x\right)$. If *m* is the mass of one end mirror, for a measurement time $t_{meas}$, this minimum momentum implies a displacement of:

(4) $$\Delta x\left(t_{meas}\right)\;=\;\frac{\Delta p}{m}\times t_{meas}\;=\;\frac{\hbar }{2\Delta x}\times \frac{t_{meas}}{m};$$

from which we extract the minimum fluctuation:

(5) $$\Delta x\left(t_{meas}\right)\;=\;\sqrt{\frac{\hbar t_{meas}}{2m}}.$$

This is the quantum noise limit of the interferometer, not of the intracavity phase sensing! The solution to determine the intrinsic noise limit in intracavity phase interferometry is to eliminate the end-cavity interferometer, as detailed in the next section.

### 3.2. Phase Measurements

**Figure 4:**
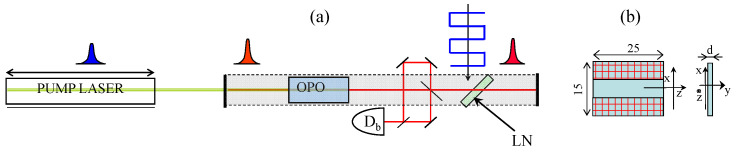
Two-pulse per cavity round-trip OPO. (**a**) sketch of the cavity configuration. The pump cavity has half the length of the OPO cavity. $D_{b}$: Beat note detector. LN: Lithium niobate modulator; (**b**) geometry of the d=0.5 mm thickness lithium niobate phase modulator put at Brewster’s angle in the OPO cavity.

Noise analysis is performed with the two-pulse per cavity OPO sketched in Figure 4a. The OPO is pumped by a Ti:sapphire laser which is mode-locked by an end-of-cavity multiple quantum well mirror, with two SF14 dispersion compensating prisms. The output of the Ti:sapphire laser is a train of 320-fs pulses at 778 nm, at a repetition rate of 160.6 MHz. The average power is 480 mW at a pump power of 6.8 W. The pump cavity has half the round-trip period of the OPO. For smaller footprint and better stability, the latter cavity has a V shape, with two curved end mirrors (radius of curvature of 3.5 m in the shorter arm; 5 m in the longer arm). The optical parametric crystal is a 5-mm-long periodically poled lithium niobate (PPLN) inserted in the cavity between two mirrors of 5 cm curvature. The output of the OPO consists therefore in two interwoven, indistinguishable, frequency combs. Since the two circulating pulses within the OPO cavity are indistinguishable, the OPO output is also a frequency comb with tooth spacing of 160.6 MHz, centered at 1.1 μm. The two pulses are extracted from the OPO cavity by a Brewster angle fused silica plate, and made to interfere via an interferometer. The total power in the beating interferometer (sum of the two non-interfering outputs) is 3.6 mW. Since the goal is to perform a phase measurement, a phase difference Δφ is introduced via a phase modulator sketched in Figure 4b. This is a lithium niobate crystal, driven by a square wave voltage synchronized to the Ti:sapphire pulse train but divided by two in frequency. The resulting modulation imprints a phase shift, alternating in sign, onto the counter circulating pulses inside the OPO cavity.

**Figure 5:**
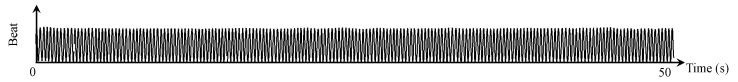
A 50-second portion of the beat note recorded by the detector $D_{b}$ in Figure 4.

**Figure 6:**
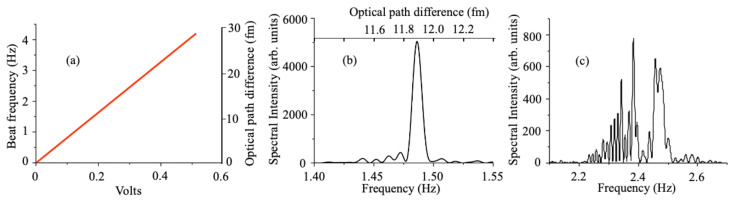
(**a**) Beat frequency versus voltage applied to the LiNbO_3_ modulator; (**b**) absolute value squared of the Fourier transform of the beat signal for a time span of 100 s, with the OPO cavity stabilized with respect to the pump cavity. The upper abscissa indicates the optical path difference corresponding to the frequency scale; (**c**) absolute value squared of the Fourier transform of the beat signal for a time span of 100 s with the stabilization turned off.

Figure 5 shows a 50-s portion of the beat note recording at 3.7 Hz, corresponding to a peak-to-peak voltage on the modulator of 0.35 V. The good visibility of the fringes is the consequence of the property of frequency combs that the tooth spacing is rigorously constant [3,4,5], and of the correlation between the interwoven optical combs that are being interfered. For the data presented in Figure 5, neither the pump laser nor the OPO cavity are stabilized, resulting in optical frequency fluctuations of each frequency comb in the tens of MHz. However, Figure 6a shows that the *difference* between the optical frequencies can be sub-Hertz, indicating the absence of the type of coupling that results in a dead band in laser gyroscopes. After locking the OPO cavity length to that of the pump laser (see Section 3.3), the Fourier transform of the time recording for a duration of 100 s at 1.5 Hz has a bandwidth of 0.01 Hz. The beat frequency is given by $\Delta \nu =\nu \Delta L/L=\Delta \varphi /(2\pi \tau_{p})$, where $\tau_{p}=6.2$ ns is the time difference between two successive OPO pulses at the phase velocity. The beat frequency scale in Figure 6b has been converted into an optical path difference in the top of the figure. A Full Width at Half Maximum (FWHM) of 0.01 Hz implies a resolution of 0.4 nanoradian. The corresponding change in optical path is LΔν/ν≈0.07 fm. In a prior application of IPI to magnetometry, a beat note bandwidth of 1 Hz was reported [9]. The narrowest beat note bandwidth was 0.17 Hz, in an application of IPI to measure the nonlinear index of lithium niobate [12]. It should be noted that the Fourier transform displayed in Figure 3 of [12] has much broader fluctuations below the half width mark than the data presented here. These fluctuations indicate a much larger mean square deviation of the phase, a parameter discussed in Section 3.4.

### 3.3. Noise Management

**Figure 7:**
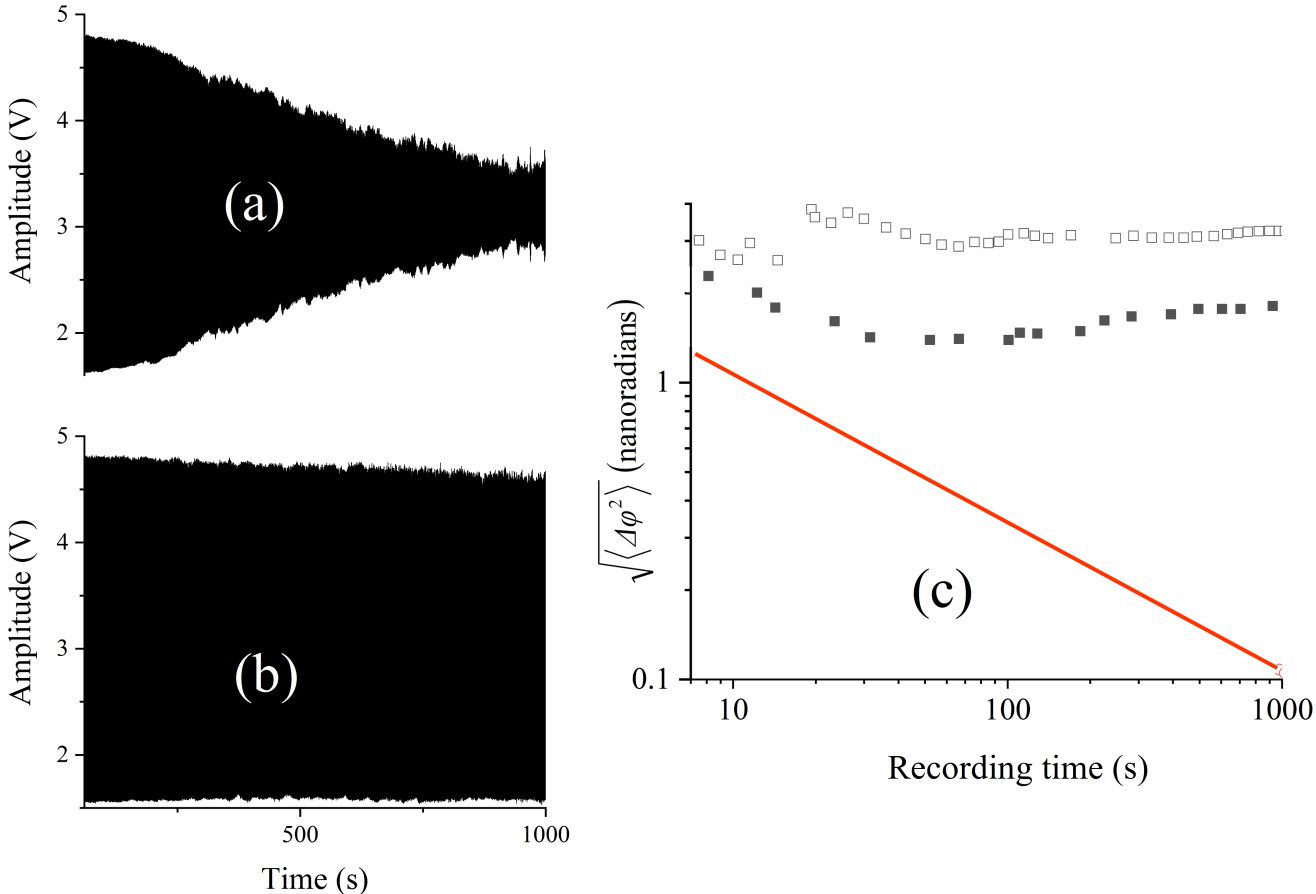
Beat note analysis. The same data-sets were used as for Figure 6. (**a**) beat note recording for 1000 s. There is no electronic stabilization. The square wave applied to the LiNbO_3_ has an amplitude of 0.25 V, resulting in a beat note of 2.25 Hz. The OPO power is 3.66 mW; (**b**) beat note recording for 1000 s. The stabilization has been turned on. The square wave applied to the LiNbO_3_ has an amplitude of 0.175 V, resulting in a beat note of 1.5 Hz. The OPO power is 3.66 mW; (**c**) mean square deviation of the phase difference seen by the two intracavity OPO pulses versus measurement time $t_{meas}$. The hollow squares correspond to the unstabilized case (**a**); the full squares to the stabilized case (**b**); the red line shows the standard quantum limit.

It can be verified that the Fourier transform of a pure sine wave at 1.5 Hz has the same linewidth as that presented in Figure 6b. In the laser-OPO configuration, relative fluctuations in the pump cavity length as compared to half the OPO cavity length result in fluctuations in the optical frequency of the OPO in order to maintain synchronism. These fluctuations result in variations of the beat note, since the beat frequency is proportional to the the OPO light frequency. A simple stabilization system consists of placing a quadrant detector after a dispersive prism to provide an error signal. The signal from the quadrant detector is fed back to a piezoelectric translator positioning an end mirror of the pump cavity [15]. A different approach was used here, which is to take the heterodyne mixing of the two pulse trains (Ti:sapphire pump and OPO) as an error signal. Both signals at 160 MHz are converted to a sine wave by low pass filters before being sent to an RF-mixer (Hewlett Packard model 10514A). The IF signal from the mixer is amplified and sent to a piezo-element controlling the Ti:sapphire cavity length. Figure 6c shows an example of beat note recording in the unstabilized case, as compared to a beat note recorded when the phase stabilization is used (Figure 6b). The piezo transducer driving an end mirror of the Ti:sapphire cavity had resonance frequencies at 300 Hz and 800 Hz. To prevent oscillation of the system, the gain of the feedback amplifier decreases approximately 1db/octave to unity gain at 1 kHz. Therefore, the stabilization is mostly effective at eliminating drift between the cavities, as illustrated by a comparison of the beat note recordings of Figure 7a,b.

**Figure 8:**
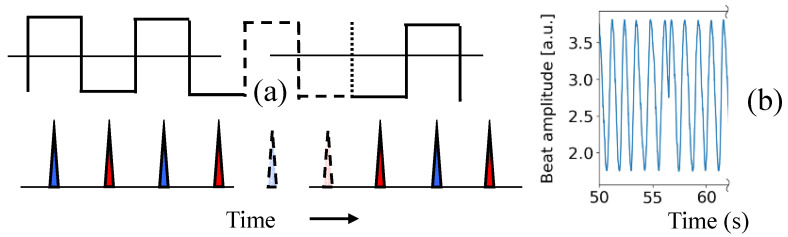
(**a**) Top: square wave applied to the phase modulator. Bottom: pulse sequence in the OPO, with the blue pulses experiencing a positive phase shift, the red ones a negative one. Dashed lines indicate a break in the triggering of the square wave. If the trigger of the square wave is interrupted for an odd number of pulses (dashed lines), the phase of the square will experience a shift of π; (**b**) a random occurrence of phase flip is shown.

Inspection of the time recording of the beat note reveals occasional phase reversals. These are due to the fact that the square wave applied to the LiNbO_3_ modulator is at half the repetition frequency of the synchronized pump and signal pulses (Figure 8). The positive half of the square wave shown on top of Figure 8a imparts a positive phase shift Δφ/2 to every other OPO signal pulse (marked in blue in the figure). The negative half-wave marks the signal pulse with a phase shift −Δφ/2. If the trigger sequence is interrupted for an odd number of pulses (dashed lines in Figure 8a), the phase of the square wave will be flipped when the pulse sequence resumes, as indicated in the figure. The result is a π phase jump in the beat note. One of these random occurrences is shown in Figure 8b. Solutions to this problem include a more robust trigger of the divider by 2, and ensuring continuity of the mode-locked pulse train, by working in a dust- and fly-free environment. Other sources of noise that contribute to the departure from the standard quantum limit are amplitude drift, noise, and jitter of the signal applied to the modulator.

A last trivial source of noise is the beat note interferometer, in which vibrations and air turbulence can cause a slight distortion of the sinusoidal beat signal. Solutions include higher mechanical stability, placing the interferometer in vacuum, and choosing to extract the output closest to the intracavity pulse crossing point to minimize the size of the detection interferometer.

### 3.4. Intrinsic Noise Limit in the Phase Measurement

The phase difference between the two intracavity pulses at each round-trip is giving rise to a beat note at a frequency $\Delta \varphi /(2\pi \tau_{p})$. The statistic of the phase errors between the two pulses creates different frequency components. Thus, taking the RMSD of the Fourier spectrum of the beat note and multiplying by $\left(2\pi \tau_{p}\right)$ gives the true RMSD of the phase. One should not confuse the phase of the beat note, with the phase difference applied between the two intracavity pulses at each round-trip.

The experimental value of the mean square deviation of the measured phase between successive pulses is:

(6) $$\sqrt{\langle \Delta \varphi^{2}\rangle }=\frac{2\pi \tau_{p}\int_{0}^{t_{meas}}\left(\Delta \nu_{b}^{2}-{\langle \Delta \nu_{b}\rangle }^{2}\right)I\left(\Delta \nu_{b}\right)d\Delta \nu_{b}}{\int_{0}^{t_{meas}}I\left(\Delta \nu_{b}\right)d\Delta \nu_{b}}.$$

The experimental values of $\sqrt{\langle \Delta \varphi^{2}\rangle }$ are plotted as a function of $t_{meas}$ in Figure 7c. For all practical purposes, the experimentalist will consider the FWHM of the beat note spectrum to be the resolution limit. However, the mean square deviation of the beat note spectrum gives a more complete measure of the noise, since it results from an integral over the spectrum (Equation (6)), rather than the spectral values near the peak. Furthermore, it allows direct comparison with the standard quantum limit, as detailed below.

One can easily estimate the quantum limit of the noise in Intracavity Phase Measurement, by using the uncertainty relation between phase and photon number $\langle \Delta N^{2}\rangle \langle \Delta \varphi^{2}\rangle \;\geq \;\frac{1}{4}$. Inside that OPO cavity, ${\langle \Delta \varphi^{2}\rangle }_{min}=\frac{1}{4\langle \Delta N^{2}\rangle }=\frac{1}{4\langle N\rangle }$, assuming a Poisson photon statistic. 〈N〉 being very large, the contribution from the OPO to the minimum phase fluctuations $\langle \Delta \varphi^{2}\rangle$ is negligible. The frequency measurement occurs in the beat note detection interferometer, where the number of photons is considerably less. In this interferometer, the number of photons accumulated after a measurement time $t_{meas}$ is $\langle N\rangle \approx P_{0}t_{meas}/\left(h\nu \right)$, where $P_{0}$ is the optical power in the interferometer. The standard quantum limit of the phase uncertainty is thus:

(7) $$\sqrt{\langle \Delta \varphi^{2}\rangle }=\frac{1}{2\sqrt{\langle N\rangle }}.$$

## 4. Conclusions

We showed that, despite of their short coherence length, ultrashort pulses can be used for the precise measurement of physical quantities acting on the phase of these pulses. An optical parametric oscillator (OPO) was pumped by 320 fs pulses from a Ti:sapphire laser with a repetition rate that corresponds to half the length of the OPO cavity. Thus, two pulses are concurrently circulating in the OPO. They are extracted at a location close to their crossing point and the beat note between the two is recorded. The two pulse trains can be considered two interwoven frequency combs. Because these two combs are created in the same active cavity at some nanosecond time interval, they are correlated and a sub-Hz beat note is routinely measured.

The sensitivity and precision of the method can be easily judged by the fact that an electric field of less than 1 V/cm on a lithium niobate crystal caused a phase shift in one of the pulses leading to a few-Hertz beat note with a bandwidth of 10 mHz. In addition, the root-mean-square deviation of the measured phase over measuring time (similar to the Allan variance) implies a remarkable low-frequency performance.

Further improvements of the setup can be stabilization of the utilized lasers, increase of the OPO power, working at a shorter OPO wavelength, and squeezing of the phase. In addition, introduction of large resonant dispersion (such as a Gires–Tournois interferometer) into the OPO cavity can significantly amplify the frequency difference between the two circulating pulses [16].

## Data Availability

The data presented in this study are available on request from the corresponding author.
